# Supplementary material for: Venomix: a simple bioinformatic pipeline for identifying and characterizing toxin gene candidates from transcriptomic data
Source: PeerJ. 2018 Jul 31;6:e5361. doi: 10.7717/peerj.5361 (PMC6074769; doi:10.7717/peerj.5361)
Supplement: Supplemental Information 3 [file peerj-06-5361-s003.gz › FinalOutput_E-6/Disintegrin_EMF10B_17/finaltree.pdf]

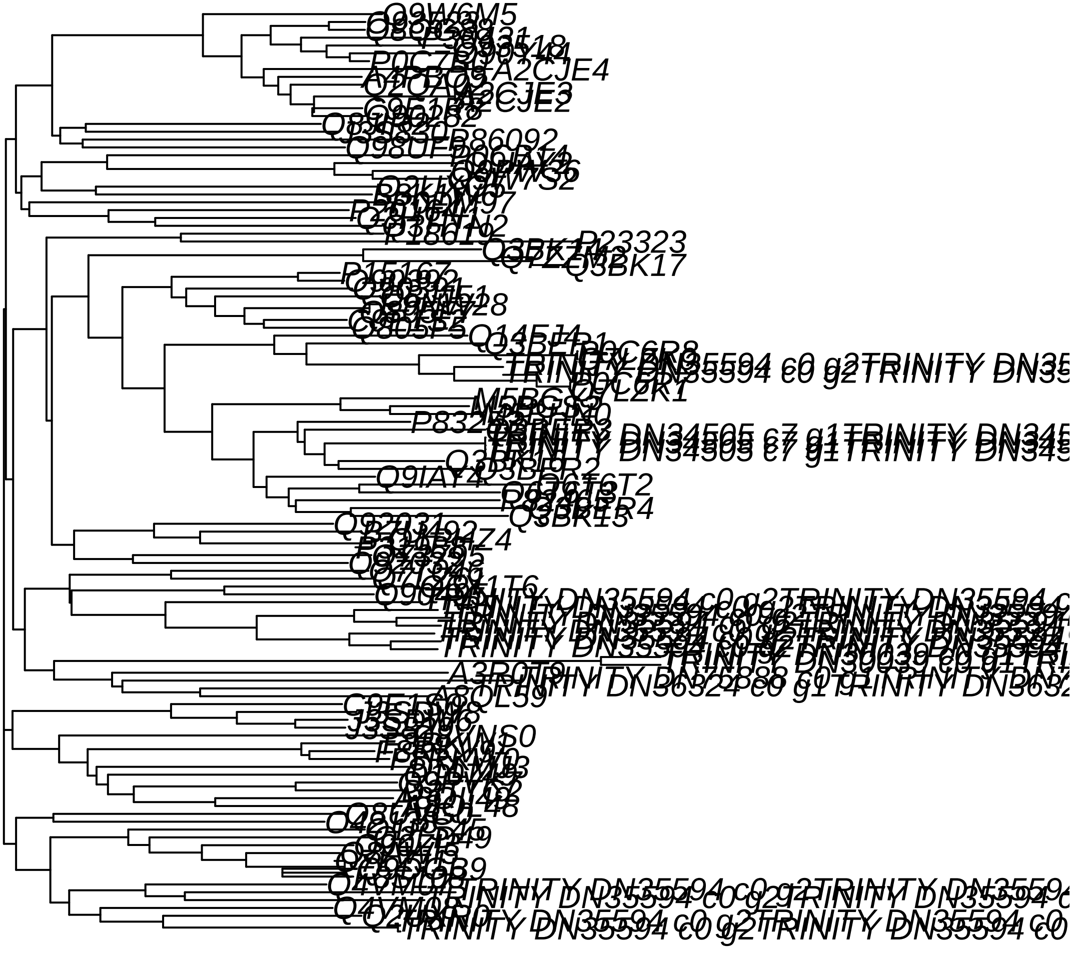
Q9W6M5
A2CJE4
P86092
P23323
Q3BK17
Q14F14
Q9IAY4
Q3BK15
A3R0T9
A8QL59
F8KYN0
Q8CH23
